# The effect of an exercise program in pregnancy on vitamin D status among healthy, pregnant Norwegian women: a randomized controlled trial

**DOI:** 10.1186/s12884-019-2220-z

**Published:** 2019-02-20

**Authors:** Miriam K. Gustafsson, Pål R. Romundstad, Signe Nilssen Stafne, Anne-Sofie Helvik, Astrid Kamilla Stunes, Siv Mørkved, Kjell Åsmund Salvesen, Per Medbøe Thorsby, Mats Peder Mosti, Unni Syversen

**Affiliations:** 1 Department of Public Health and Nursing, Faculty of Medicine and Health Sciences, Norwegian University of Science and Technology (NTNU), PO Box 8905, 7491 Trondheim, Norway; 2 Division of Mental Health Care, Trondheim University Hospital (St. Olavs hospital), Trondheim, Norway; 3 Clinic of Clinical Services, Trondheim University Hospital (St. Olavs hospital), Trondheim, Norway; 4 Trondheim University Hospital (St. Olavs hospital), Trondheim, Norway; 5 Department of Clinical and Molecular Medicine, Faculty of Medicine and Health Sciences, Norwegian University of Science and Technology (NTNU), Trondheim, Norway; 6 Department of Obstretics and Gynaecology, Trondheim University Hospital (St. Olavs hospital), Trondheim, Norway; 7 Hormone Laboratory, Department of Medical Biochemistry, Oslo University Hospital, Aker sykehus, Oslo, Norway; 8 Department of Endocrinology, Trondheim University Hospital (St. Olavs hospital), Trondheim, Norway

**Keywords:** Bioavailable 25(OH)D, Exercise, Free 25(OH)D, Physical activity, Pregnancy, RCT, Serum 25(OH)D

## Abstract

**Background:**

Vitamin D insufficiency is common in pregnant women worldwide. Regular prenatal exercise is considered beneficial for maternal and fetal health. There is a knowledge gap regarding the impact of prenatal exercise on maternal vitamin D levels.

The objective of this study was to investigate whether a prenatal exercise program influenced serum levels of total, free and bioavailable 25-hydroxyvitamin D (25(OH)D) and related parameters. This is a post hoc analysis of a randomized controlled trial with gestational diabetes as the primary outcome.

**Methods:**

Healthy, pregnant women from two Norwegian cities (Trondheim and Stavanger) were randomly assigned to a 12-week moderate*-*intensity exercise program (Borg perceived rating scale 13–14) or standard prenatal care. The intervention group (*n* = 429) underwent exercise at least three times weekly; one supervised group training and two home based sessions. The controls (*n* = 426) received standard prenatal care, and exercising was not denied. Training diaries and group training was used to promote compliance and evaluate adherence. Serum levels of 25(OH)D, parathyroid hormone, calcium, phosphate, magnesium and vitamin D-binding protein were measured before (18–22 weeks′ gestation) and after the intervention (32–36 weeks′ gestation). Free and bioavailable 25(OH)D concentrations were calculated. Regression analysis of covariance (ANCOVA) was applied to assess the effect of the training regime on each substance with pre-intervention levels as covariates. In a second model, we also adjusted for study site and sampling month. Intention-to-treat principle was used.

**Results:**

A total of 724 women completed the study. No between-group difference in serum 25(OH)D and related parameters was identified by ANCOVA using baseline serum levels as covariates. The second model revealed a between-group difference in levels of 25(OH)D (1.9, 95% CI 0.0 to 3.8 nmol/L; *p* = 0.048), free 25(OH)D (0.55, 95% CI 0.10 to 0.99 pmol/L; *p* = 0.017) and bioavailable 25(OH)D (0.15 95% CI 0.01 to 0.29 nmol/L; *p* = 0.036). No serious adverse events related to regular exercise were seen.

**Conclusion:**

This study, a post hoc analysis, indicates that exercise may affect vitamin D status positively, and emphasizes that women with uncomplicated pregnancies should be encouraged to perform regular exercise.

**Trial registration:**

ClinicalTrials.gov: NCT00476567, registered May 22, 2007.

**Electronic supplementary material:**

The online version of this article (10.1186/s12884-019-2220-z) contains supplementary material, which is available to authorized users.

## Background

Pregnancy represents a unique metabolic state with adaptive physiological changes including the vitamin D endocrine system [1–4]. Circulating 25-hydroxyvitamin D (25(OH)D) is a measure of vitamin D status, and a pre-hormone for the active form, *1,25-dihydroxyvitamin D* (1,25(OH)_2_D), which is mainly synthesized in the kidneys [1, 2]. Most circulating 25(OH)D and 1,25(OH)_2_D bind to vitamin D-binding protein (DBP) and albumin [5, 6]. In pregnancy, a pronounced rise in maternal 1,25(OH)_2_D concentration ensures increased calcium absorption and mineralization of the fetal skeleton [2, 3]. This increment is dependent on sufficient 25(OH)D [4]. Hypovitaminosis D is frequent among pregnant women and has been linked to negative health consequences for both the mother and child including pre-eclampsia, rickets, osteoporosis, and cardiovascular disease (CVD) [1–4, 7].

Vitamin D affects muscle directly by binding of 1,25(OH)_2_D to the vitamin D receptor (VDR) and indirectly through the calcium and phosphate balance [8]. Physical activity is reported to increase 25(OH)D levels, however, this has been proposed to be attributed to solar ultraviolet B (UV-B) radiation [9–11]. Yet, a positive association has also been observed between indoor physical activity and 25(OH)D levels [10, 11]. Data from intervention studies on the effects of long-term exercise on vitamin D status are scarce [9, 12].

The American College of Obstetrics and Gynecologists (ACOG) recommends women with uncomplicated pregnancies to exercise on moderate intensity for at least 20–30 min most days of the week [13]. The impact of prenatal exercise on vitamin D has, however, been little explored [12, 14, 15].

Therefore, based on a randomized controlled trial (RCT) of 855 pregnant women, designed to investigate health effects of exercise, we aimed to do a post hoc analysis to explore a potential relation between regular exercise in pregnancy and the vitamin D endocrine system.

## Methods

### Study design and participants

Authors of this study conducted a two-armed, two-center RCT, and health effects of a 12-week exercise program during pregnancy was compared with standard prenatal care [16]. Gestational diabetes was the primary outcome [16]. Information was collected that enabled a post hoc analysis to assess the effects of regular exercise on vitamin D levels and related parameters.

Between April 2007 and June 2009 in Trondheim and October 2007 and January 2009 in Stavanger, pregnant women attending the 18-week routine ultrasound were enrolled [16]. Eligible women were healthy Caucasian, 18 years or older with a singleton live fetus. In accordance with ACOG, exclusion criteria were pregnancy complications, high risk for preterm delivery or diseases that could hinder participation [13, 16]. Women living far from the hospital were excluded (Additional file 1, study protocol). Clinical data and blood samples were collected before and after the intervention (gestational weeks 18–22 and 32–36, respectively).

The study was approved by the Regional Committee for Medical and Health Research Ethics (REK 4.2007.81) and performed in accordance with the Declaration of Helsinki. The trial is registered in the ClinicalTrials.gov (NCT 00476567).

### Randomization and masking

The women received information about the study, and gave informed written consent [16]. Concealed randomization in blocks of 30, using a digital computer technique was performed. The personnel involved in the exercise-program and outcome assessment had no influence [16]. Masking of participants and study investigators to group allocation was not possible.

### Intervention procedures

The intervention group was provided a 12-week standardized exercise program, including both aerobic and strength training (20–36 weeks′ gestation), in line with ACOG and the Norwegian National Report on Physical Activity and Health [13, 16]. Group exercise sessions of 60 min, led by a physiotherapist, were offered once a week. Additionally, the women were encouraged to exercise at home at least twice weekly [16].

The controls received standard prenatal care and customary information by midwife or general practitioner, and exercising was not denied. Both groups received written recommendations on diet, pelvic floor muscle exercises and pregnancy-related lumbopelvic pain [16]. In both groups, questionnaires were used before and after intervention to assess physical activity. Exercise intensity was measured by the Borg rating of perceived exertion (RPE) scale (score 6–20), and level of moderate physical intensity (score 13–14), in accordance with ACOG [13, 16]. Training diaries and group training was used to promote compliance and evaluate adherence, which was defined as exercising 3 days weekly or more at moderate intensity.

A self-administrated optical mark readable Food Frequency Questionnaire, containing around 180 food items was used before and after the intervention to obtain information about vitamin D and calcium [17].

### Serum analyses and calculation of free and bioavailable 25(OH)D

Fasting blood samples were drawn before and after the intervention [18]. The following analyses were performed at Trondheim University Hospital: 25(OH)D and parathyroid hormone (PTH) by electrochemiluminescence immunoassay (ECLIA), calcium by a colorimetric method, and phosphate, magnesium, albumin and creatinine by photometric methods. All assays were delivered by Roche Diagnostics Ltd., Switzerland. Total calcium was corrected for albumin concentration. Vitamin D-binding protein (DBP) was analyzed at the Hormone Laboratory, Oslo University Hospital by an in-house competitive radioimmunoassay with GC-globulin (Sigma-Aldrich Corp, St. Louis, MO, USA) and polyclonal anti-GC-globulin antibodies (DakoCytomation, Glostrup, Denmark). Reference range, limit of detection and coefficient of analytical variation (CV) for the different analyses are presented in Additional file 2.

An equation developed by Bikle et al. was applied for determination of free 25(OH)D [5]. Bioavailable 25(OH)D was calculated as the sum of albumin-bound and free 25(OH)D (Additional file 3) [6].

### Outcomes

The main outcome was the effects of exercise in pregnancy on total, free and bioavailable 25(OH)D. Secondary outcomes were effects on PTH, total and corrected calcium, magnesium, phosphate, and DBP.

### Statistical analyses

The analysis was performed according to the intention-to-treat (ITT) principle, and the approach to handling missing data was complete case analysis. SPSS statistics Version 24.0 (Armonk, NY: IBM Corp) and Stata version 13 (StataCorp LP, College Station, TX, USA) were applied. The power calculation and sample size estimation were done for the primary outcome, gestational diabetes [16]. Few experimental studies have investigated the effects of exercise on young women, and so far, no RCTs have addressed the vitamin D response to an exercise program in pregnancy [15, 19]. The power calculation in the present study was based on a study exploring the effect of short-time exercise on 25-hydroxyvitamin D in young women [19]. In the present study, a sample size of 772 (386 in the intervention group and 386 in the control group) conferred 80% power with two-sided *p* = 0.05, to detect a between-group difference of 5 nmol/L in 25(OH)D levels.

Regression analysis of covariance (ANCOVA) was used to assess the effect of the training regime on each substance, with pre-intervention levels as covariates. In a second model, we also adjusted for study site and sampling month. We performed a sensitivity analysis using a mixed-effects model with random slope for 25(OH)D. The estimates were similar, and therefore only estimates from ANCOVA are presented.

## Results

### Participants

**Fig. 1:**
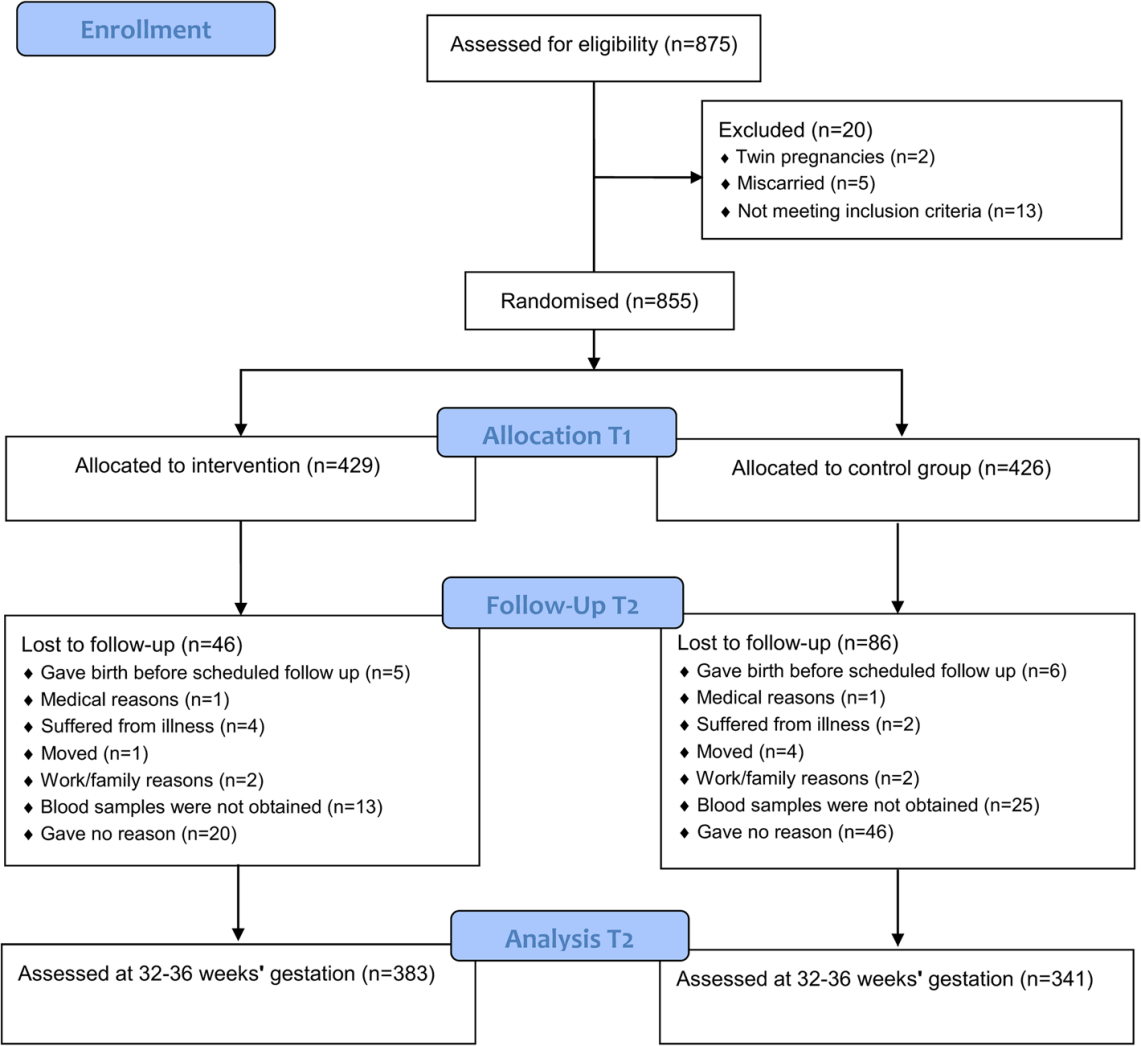
Consolidated Standards of Reporting Trials (CONSORT) flowchart

A total of 875 pregnant women were assessed for eligibility [16]. Twenty women were excluded, and 855 were randomized into either the intervention or control groups (Fig. 1). A total of 724 women (85%) completed the study. Loss to follow-up was 15%: 46 of 429 (11%) in the intervention group, and 86 of 426 (20%) in the control group. No serious adverse events related to regular exercise were seen, and nobody withdrew due to adverse events.

Participant characteristics are presented in Table 1, and baseline serum levels of vitamin D and related parameters in Table 2. Pre-pregnancy body mass index (BMI) was 23.0 in the intervention group and 23.3 among the controls. According to classification by the World Health Organization, both groups had normal BMI [20]. Any minor differences in baseline characteristics between the groups were within the expected limits for random allocation. In the intervention group, 214 (50%) participants adhered to the exercise program.

**Table 1:** Characteristics of the study population at inclusion

	Intervention group (*n* = 429)	Control group (*n* = 426)
Age (years)	30.5 ± 4.4	30.4 ± 4.3
Weight (kg)^a^	70.4 ± 9.8	70.8 ± 10.3
Body Mass Index (kg/m^2^)^a^	24.7 ± 3.0	25.0 ± 3.4
Gestational week at baseline sampling	20.3 ± 1.5	20.3 ± 1.7
Marital status		
Married/cohabitant^b^	413 (96.3)	421 (98.8)
Single	15 (3.5)	5 (1.2)
Education level		
Elementary school	3 (0.7)	2 (0.5)
High School	39 (9.1)	51 (12.0)
University	387 (90.2)	373 (87.6)
Paid work or self-employed^a^	405 (94.4)	393 (92.3)
Parity		
0	247 (57.6)	239 (56.1)
1	125 (29.1)	129 (30.3)
2	45 (10.5)	45 (10.6)
≥ 3	12 (2.8)	13 (3.0)
Smoking	7 (1.6)	2 (0.5)
State of health		
Excellent or good	384 (89.5)	376 (88.3)
Fair	40 (9.3)	45 (10.6)
Poor	5 (1.2)	5 (1.2)
Exercise regularly	228 (53.1)	216 (50.7)

Continuous variables are given as means ± standard deviations (SD), and categorical variables are given as numbers (*n*) with percentages (%)

^a^One is missing in the control group

^b^One is missing in the intervention group

**Table 2:** Vitamin D and related parameters at inclusion

	Intervention group (*n* = 429)	Control group (*n* = 426)
25(OH)D (nmol/L)^a^	65.9 ± 25.9	66.2 ± 23.7
Free 25(OH)D (pmol/L)^b^	15.3 ± 6.1	15.2 ± 5.8
Bioavailable 25(OH)D (nmol/L)^b^	5.1 ± 2.1	5.1 ± 1.9
PTH (pmol/L)^a^	2.7 ± 1.1	2.9 ± 1.1
Calcium (mmol/L)^a^	2.27 ± 0.07	2.27 ± 0.07
Corrected calcium (mmol/L)^a^	2.34 ± 0.06	2.33 ± 0.06
Phosphate (mmol/L)^a^	1.19 ± 0.12	1.19 ± 0.12
Magnesium (mmol/L)^b^	0.75 ± 0.05	0.75 ± 0.04
Vitamin D-binding protein (μmol/L)^b^	5.7 ± 0.8	5.8 ± 0.8
Albumin (g/L)^a^	36.8 ± 2.1	36.6 ± 2.0
Daily vitamin D intake (μg)	10.1 ± 6.6	10.8 ± 7.3
Daily Calcium intake (mg)^c^	962.1 ± 350.0	987.6 ± 397.0
Women with 25(OH)D level < 50 nmol/L^a^	125 (29.1)	107 (25.1)

Serum levels are presented as means ± standard deviations (SD), and categorical variables are given as numbers (*n*) with percentages (%)

^a^ One is missing in the control group

^b^ Two are missing in the control group

^c^One is missing in the intervention group and two are missing in the control group

**Fig. 2:**
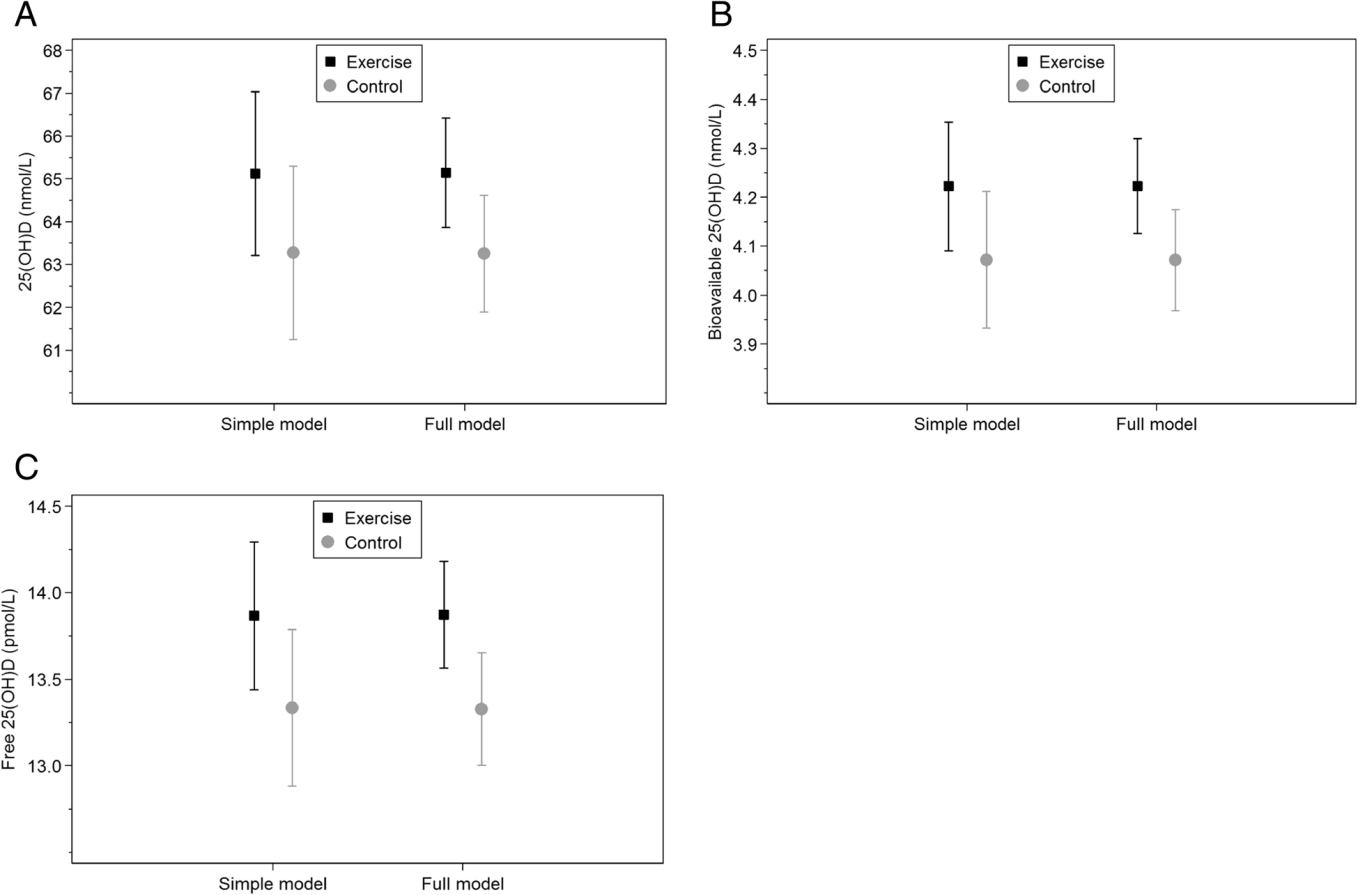
Serum levels of vitamin D after a prenatal 12-week exercise program (20–36 weeks′ gestation). The filled square represents the exercise group and the grey circle represents the control group. Regression analysis of covariance (ANCOVA) was used. Simple model, adjusted for baseline serum level for each substance. Full model, simple model + study site and sampling month. **a** Total 25(OH)D (nmol/L). **b** Bioavailable 25(OH)D (nmol/L). **c** Free 25(OH)D (pmol/L)

After adjusting for baseline concentrations of each substance, the ITT analysis showed no significant effect of the exercise program on levels of total, free and bioavailable 25(OH)D and related substances. In a second model we additionally adjusted for study site and sampling month, and revealed a significant between-group difference in serum levels of 25(OH)D (1.9, 95% confidence interval (CI) 0.0 to 3.8 nmol/L; *p* = 0.048), free 25(OH)D (0.55, 95% CI 0.10 to 0.99 pmol/L; *p* = 0.017) and bioavailable 25(OH)D (0.15, 95% CI 0.01 to 0.29 nmol/L; *p* = 0.036). PTH, corrected calcium, phosphate, magnesium, DBP and albumin did not differ between groups. Both statistical models showed similar effect estimates, but the 95% CI was narrower in adjusted model. The results are presented in Table 3 and Fig. 2.

**Table 3:** Estimates of the intervention effects after a prenatal exercise programme among Caucasian pregnant women

Simple model	Difference between the groups	95% CI	*p*-value^a^
25(OH)D (nmol/L)	1.9	−0.9 to 4.6	0.194
Free 25(OH)D (pmol/L)	0.53	−0.09 to 1.15	0.093
Bioavailable 25(OH)D (nmol/L)	0.15	−0.04 to 0.34	0.123
PTH (pmol/L)	0.15	−0.03 to 0.33	0.102
Calcium (mmol/L)	−0.001	−0.009 to 0.007	0.880
Corrected calcium (mmol/L)	0.000	−0,007 to 0,008	0.900
Phosphate (mmol/L)	−0.001	−0.019 to 0.016	0.863
Magnesium (mmol/L)	−0.001	−0.006 to 0.004	0.700
DBP (μmol/L)	−0.08	−0.17 to 0.02	0.106
Albumin (g/L)	−0.08	−0.29 to 0.13	0.428
Full model	Difference between the groups	95% CI	*p*-value^b^
25(OH)D (nmol/L)	1.9	0.0 to 3.8	0.048
Free 25(OH)D (pmol/L)	0.55	0.10 to 0.99	0.017
Bioavailable 25(OH)D (nmol/L)	0.15	0.01 to 0.29	0.036
PTH (pmol/L)	0.16	−0.02 to 0.33	0.08
Calcium (mmol/L)	−0.001	- 0.009 to 0.007	0.773
Corrected calcium (mmol/L)	0.000	−0.008 to 0.008	0.985
Phosphate (mmol/L)	−0.003	−0.020 to 0.014	0.731
Magnesium (mmol/L)	−0.0004	−0.0051 to 0.0043	0.865
DBP (μmol/L)	−0.09	−0.18 to 0.01	0.067
Albumin (g/L)	−0.08	-0.29 to 0.13	0.447

Regression analysis of covariance (ANCOVA) was used

^a^Simple model, adjusted for baseline serum level for each substance

^b^Full model, Simple model + study site and sampling month

## Discussion

To the best of our knowledge, this is the first study to assess the effects of an exercise program during pregnancy on circulating vitamin D levels. After adjustment for relevant covariates, we observed higher levels of total, free and bioavailable 25(OH)D in the exercise group. The study design, the large sample size and the statistical modeling reduced the risk of bias. Regular exercise during pregnancy is recommended due to health benefits. This is supported by our data which suggest that exercise in pregnancy may affect vitamin D status positively.

### The effects of prenatal exercise on vitamin D and related parameters

We observed a between-group difference in 25(OH)D levels of 2 nmol/L after long-term exercise during pregnancy. Several studies have explored the impact of exercise on vitamin D in the non-pregnant state [9, 12, 19, 21]. Levels of 25(OH)D increased by 21.5 nmol/L (8.6 ng/mL) in elderly individuals executing a 8-week aerobic program in combination with antioxidant supplementation [22]. The influence of seasonal variation was, however, uncertain, due to lack of controls. In contrast, no effects on 25(OH)D levels were seen among non-pregnant Finnish women after a 12 months impact exercise program [21]. A high proportion of lost to follow-up and low compliance may have affected the results [21]. Data on the effects of short-term exercise on vitamin D are diverging [12, 19]. A rise in 25(OH)D was reported after a bicycling endurance exercise session among young, healthy Japanese men and women [19]. After 24 h, mean 25(OH)D level among women was 5.3 nmol/L higher than at baseline [19]. In the current study, the between-group difference in 25(OH)D was modest compared with the Japanese study. The discrepancy may be due to differences in exercise type and length, mean basal levels of 25(OH)D, analytical methods and the pregnant state [9, 19, 21]. Moreover, ITT analysis was performed in the present RCT, and it is reasonable the large number of noncompliant women (50% in the intervention group) contributed to diluted results. A novelty of the present study lies in the assessment of free and bioavailable 25(OH)D. It has been proposed that these are important biomarkers for vitamin D status in the pregnant state since the DBP concentration alters [5, 6, 23]. A proportionally larger between-group difference was observed in free 25(OH)D compared to total concentration. In accordance with observations in the non-pregnant state, DBP levels were unaffected by exercise [19].

We observed no effects on PTH, calcium, phosphate, magnesium and albumin levels. Previous reports concerning the impact of exercise on PTH and calcium are conflicting, and research on pregnant women are lacking [12, 19, 21, 22].

### Mechanisms for exercise-induced changes in the vitamin D endocrine system

There may be several mechanisms for the exercise-induced rise reported both in 25(OH)D and 1,25(OH)_2_D levels, however, they are not fully understood, and it is unknown if they differ between the pregnant and nonpregnant state [12, 19]. Firstly, 25(OH)D may be mobilized from skeletal muscle during exercise. Muscle cells contain a reservoir of 25(OH)D, and can accrue and return the vitamin to the extracellular space [24]. Regular exercise may increase muscle mass, thus providing a larger pool of 25(OH)D, which can be mobilized. This may be beneficial during pregnancy as the substantial rise in 1,25(OH)_2_D concentration is dependent on sufficient 25(OH)D [4]. A rise in circulating 1,25(OH)_2_D is suggested to block the muscle cells′ ability to store 25(OH)D, thereby facilitating release [24]. Accordingly, more circulating 25(OH)D is available for 1,25(OH)_2_D synthesis [24]. The effects of vitamin D are mediated through 1,25(OH)_2_D, exerting genomic and nongenomic actions via VDR in muscle cells [8, 25, 26]. Increased VDR expression was found in rodents after a bout of resistance exercise, but not after endurance exercise [25]. Additionally, intramuscular expression of cytochrome P450 27B1, the enzyme converting 25(OH)D to 1,25(OH)2D, was higher in rats performing resistance exercise compared with controls [1, 25]. Sixteen weeks vitamin D supplementation has also been shown to enhance VDR gene expression in skeletal muscle in older women [26]. We did not obtain muscle biopsies from our participants, and therefore expression of VDR and cytochrome P450 27B1 could not be assessed.

Adipose tissue is another potential source for the exercise-induced rise in vitamin D [27]. It is claimed that the vitamin is stored and sequestered in adipose tissue leading to less availability [27]. This suggest that obese people exhibit a more modest response in 25(OH)D due to exercise, however, this needs to be confirmed. A weight-loss program in overweight and obese women resulted in higher serum 25(OH)D, indicating that a reduction in fat mass increases the access [27]. Our participants had a normal pre-pregnancy BMI, which may imply a more pronounced vitamin D response compared to obese women.

Finally, synthesis and release of 25(OH)D from the liver could be increased due to exercise. However, data on this topic are lacking. A rat study, addressing the effects of long-term exercise, showed that degradation of 25(OH)D may be reduced [28]. Higher 24,25(OH)_2_D levels were observed among immobilized rats compared to the exercise group and controls, implying that physical activity prevents catabolism of 25(OH)D [28]. It is unknown if this translates to humans, thereby contributing to higher vitamin D status by exercising. Measurement of 24,25(OH)_2_D levels is warranted in future studies.

Some studies have shown an increment in circulating 1,25(OH)_2_D after exercise [12, 22]. This could be attributed to a temporary decrease in ionized calcium, as well as phosphate, followed by a rise in PTH, which stimulates 1,25(OH)_2_D production [11, 12]. We observed no between-group difference in total and corrected calcium, phosphate, magnesium and PTH levels. Due to rapid feedback mechanisms, transient changes in calcium and PTH levels may be difficult to detect in long-term exercise studies [11, 12, 21]. PTH secretion during exercise could also be stimulated by catecholamines and acidosis [12]. The heterogeneous results regarding PTH may be attributed to differences in exercise type, intensity and duration, in addition to physical fitness [12, 21]. Furthermore, the exercise-induced PTH response is dependent on the resting level [12, 21].

There is a knowledge gap concerning the impact of exercise on prenatal PTH secretion. During pregnancy, PTH declines or remains stable, and other regulators including PTH-related protein (PTHrP) may account for most of the circulating 1,25(OH)_2_D [1–3]. Further studies are needed to fully understand the complex relationship between exercise in pregnancy and alterations in the maternal vitamin D endocrine system.

### Clinical implications

Developmental origins of health and disease have gained increased attention, and maternal hypovitaminosis D during fetal life is suggested to be of significance for the risk of CVD and osteoporosis in the offspring [4, 7, 29].

Vitamin D has direct effects on skeletal muscle, and deficiency has been associated with atrophy of type II (fast-switch) fibers [8]. This is reflected in negative health effects as myopathy and muscle weakness [8, 26]. Moreover, low vitamin D is associated with fatty infiltration of the skeletal muscle independent of BMI among women, and muscle adiposity is suggested to affect muscle strength [8, 30]. In line with this, vitamin D supplementation has been shown to improve muscle strength, physical performance, balance and to reduce falls [8, 30].

Vitamin D is an important regulator of calcium homeostasis and bone metabolism [1, 21]. Weight-bearing exercise has positive effects on bone mineral density (BMD) among none-pregnant adults [31, 32]. Low 25(OH)D levels among pregnant women has been associated with reduced peak bone mass in the offspring [33]. The maternal bone turnover is reported to increase during pregnancy, and a modest decline in BMD may appear [1]. A recent trial showed that BMD loss during pregnancy was smaller among physically active compared to sedentary women [34]. However, the effects of exercise on bone remodeling in pregnancy is not elucidated.

CVD accounts for half of all deaths among European women [35]. Vitamin D is important for cardiovascular function and deficiency is negatively associated with CVD [7, 10, 11]. Vitamin D and physical activity seem to modify CVD risk, and to have synergistic beneficial effects [36]. Vitamin D deficiency in pregnancy may influence blood pressure, and the renin–angiotensin system in the offspring, resulting in increased CVD risk [7].

Given the high prevalence of vitamin D insufficiency in pregnancy worldwide, preventive strategies to avoid adverse health effects in mother and offspring are needed. The increase in vitamin D levels due to regular indoor physical activity, described in a previous observational study, was estimated to be as effective as around 5 μg vitamin D supplement intake daily [10].

### Exercise intensity and volume

Regular physical activity potentially improves cardiorespiratory fitness, usually measured in maximal oxygen uptake (VO_2_max) or metabolic equivalent tasks (METs) [37]. VO_2_max does not change during pregnancy, resting heart rate (HR) is increased, and maximal HR is slightly lower compared with post-partum [37, 38]. Moderate intensity exercise has been classified as corresponding to moderate 3–6 METs [37, 38]. This has been questioned, and it has been argued the intensity should be relative to the woman’s own maximal aerobic capacity [38]. In the present study, the exercise intensity was relative to the capacity of each participant as the Borg RPE scale was used. Although pregnant women also can monitor exercise intensity by HR, the ACOG’s guidelines, advocates the usage of the Borg RPE scale [13, 38].

### Strengths and limitations

The major strengths of the present study are the large sample size, ITT analysis, statistical modeling and the standardized procedures for sampling. However, the study has limitations: The study is a post hoc analysis of a RCT that was not designed to answer the specific research question. Although post hoc analysis is prone to data dredging bias when performing multiple unplanned analyses, we analyzed available serum from the original RCT to get necessary data to the current study [39]. Furthermore, multiple comparisons are a weakness with the post hoc analysis and increase the risk for the associations observed to be due to chance alone [40, 41]. Hence, the results should be interpreted with caution. The participants were well-educated Caucasian women with low-risk pregnancies, which may affect the generalizability. Serum 25(OH)D was analyzed by ECLIA (Roche), although liquid chromatography-tandem mass spectrometry (LC-MS/MS) is considered to be the gold standard [42]. Calculated free 25(OH)D may give an overestimation compared to direct measurement of free 25(OH)D [43]. We did not obtain data on individual sunlight exposure, and thus UV-B could be claimed to contribute to the observed differences in vitamin D levels. However, the study design, including a large number of participants, and the statistical modeling reduces the risk for bias. The loss to follow-up was 15% in the present trial. Generally around 5% loss to follow-up in a RCT is acceptable, whereas more than 20% may be a serious threat against the validity [44]. Based on the fact that in trials with lifestyle interventions, drop-out rates less than 20% are rarely achieved, Altman has proposed that one must consider the circumstances when assessing if a trial is good [45]. All in all, we cannot rule out that the attrition bias has weakened the internal validity in the present study. Only 50% in the intervention group adhered to the intervention. This is in agreement with previous studies reporting a decrease in regular exercise during pregnancy [46, 47]. In a recent Norwegian cohort study (*n* = 3482) only 15% of the pregnant women in second trimester followed the recommendations from ACOG [48]. A RCT from Brazil reported that high compliance is challenging in studies exploring the effects of prenatal exercise among pregnant women. The study investigated the effect of prenatal exercise program three times weekly for 16 weeks and reached an adherence of only 40% [49]. A future RCT, investigating the effects of regular prenatal exercise on vitamin D levels as a pre-defined primary outcome, and with a more complete adherence is warranted.

## Conclusions

Regular exercise during pregnancy is recommended due to positive health effects. This is the first RCT investigating effects of long-term exercise on vitamin D and related parameters during pregnancy. Our data indicate that exercise may affect vitamin D status positively and emphasize that women with uncomplicated pregnancies should be encouraged to perform regular exercise. However, this is a post hoc analysis and the results need confirmation in future RCTs.

## Additional files

Additional file 1: Study protocol. (PDF 61 kb)

Additional file 2: The reference range, limit of detection and total analytical coefficient of variation (CV) of biochemical methods used. (PDF 61 kb)

Additional file 3: Calculation of free and bioavailable 25(OH)D. (PDF 469 kb)

## Abbreviations

1,25(OH)_2_D: *1,25-dihydroxyvitamin D*
25(OH)D: 25-hydroxyvitamin D
ACOG: American College of Obstetrics and Gynecologists
ANCOVA: Regression analysis of covariance
BMD: Bone mineral density
BMI: Body mass index
Borg RPE scale: Borg rating of perceived exertion scale
CI: Confidence interval
CONSORT: Consolidated Standards of Reporting Trials
CV: Coefficient of analytical variation
CVD: Cardiovascular disease
DBP: Vitamin D-binding protein
ECLIA: Electrochemiluminescence immunoassay
ITT: Intention-to-treat
LC-MS/MS: Liquid chromatography-tandem mass spectrometry
MET: Metabolic equivalent task
PTH: Parathyroid hormone
PTHrP: PTH-related protein
RCT: Randomized controlled trial
RIA: Radioimmunoassay
SD: Standard deviation
UV-B: Ultraviolet B
VDR: Vitamin D receptor
VO_2_max: Maximal oxygen uptake

## References

[1] Kovacs CS (2016). Maternal mineral and bone metabolism during pregnancy, lactation, and post-weaning recovery. Physiol Rev.

[2] Moon RJ, Harvey NC, Cooper C (2015). ENDOCRINOLOGY IN PREGNANCY: influence of maternal vitamin D status on obstetric outcomes and the fetal skeleton. Eur J Endocrinol.

[3] Karras SN, Fakhoury H, Muscogiuri G, Grant WB, van den Ouweland JM, Colao AM, Kotsa K (2016). Maternal vitamin D levels during pregnancy and neonatal health: evidence to date and clinical implications. Ther Adv Musculoskelet Dis.

[4] Hollis BW, Wagner CL. Vitamin D supplementation during pregnancy: improvements in birth outcomes and complications through direct genomic alteration. Mol Cell Endocrinol. 2017. 10.1016/j.mce.2017.01.039 28188842

[5] Bikle D, Bouillon R, Thadhani R, Schoenmakers I. Vitamin D metabolites in captivity? Should we measure free or total 25(OH)D to assess vitamin D status? J Steroid Biochem Mol Biol. 2017. 10.1016/j.jsbmb.2017.01.007 PMC9005158 28093353

[6] Powe CE, Ricciardi C, Berg AH, Erdenesanaa D, Collerone G, Ankers E, Wenger J, Karumanchi SA, Thadhani R, Bhan I (2011). Vitamin D-binding protein modifies the vitamin D-bone mineral density relationship. J Bone Miner Res.

[7] Gezmish O, Black MJ (2013). Vitamin D deficiency in early life and the potential programming of cardiovascular disease in adulthood. J Cardiovasc Transl Res.

[8] Ceglia L, Harris SS (2013). Vitamin D and its role in skeletal muscle. Calcif Tissue Int.

[9] Sun X, Cao ZB, Tanisawa K, Taniguchi H, Kubo T, Higuchi M (2018). Effects of chronic endurance exercise training on serum 25(OH)D concentrations in elderly Japanese men. Endocrine.

[10] Scragg R, Camargo CA (2008). Frequency of leisure-time physical activity and serum 25-hydroxyvitamin D levels in the US population: results from the third National Health and nutrition examination survey. Am J Epidemiol.

[11] Wanner M, Richard A, Martin B, Linseisen J, Rohrmann S (2015). Associations between objective and self-reported physical activity and vitamin D serum levels in the US population. Cancer Causes Control.

[12] Maimoun L, Sultan C (2009). Effect of physical activity on calcium homeostasis and calciotropic hormones: a review. Calcif Tissue Int.

[13] ACOG Committee Opinion No**.** 650. Physical activity and exercise during pregnancy and the postpartum period. Obstet Gynecol 2015, 126(6):e135–e142. 10.1097/AOG.0000000000001214 26595585

[14] Di Mascio D, Magro-Malosso ER, Saccone G, Marhefka GD, Berghella V (2016). Exercise during pregnancy in normal-weight women and risk of preterm birth: a systematic review and meta-analysis of randomized controlled trials. Am J Obstet Gynecol.

[15] Howell CN, Hall JT, Ebeling MD, Shary JR, Baatz JE, Newton DA, Hollis BW, Wagner CL. The effect of physical activity on Vitamin D status in pregnant women participating in a randomized controlled trial. J Nutr Food Sci. 2018;8(5).

[16] Stafne SN, Salvesen KA, Romundstad PR, Eggebo TM, Carlsen SM, Morkved S (2012). Regular exercise during pregnancy to prevent gestational diabetes: a randomized controlled trial. Obstet Gynecol.

[17] Andersen LF, Veierod MB, Johansson L, Sakhi A, Solvoll K, Drevon CA (2005). Evaluation of three dietary assessment methods and serum biomarkers as measures of fruit and vegetable intake, using the method of triads. Br J Nutr.

[18] Gustafsson MK, Romundstad PR, Stafne SN, Helvik AS, Stunes AK, Morkved S, Salvesen KA, Thorsby PM, Syversen U (2018). Alterations in the vitamin D endocrine system during pregnancy: a longitudinal study of 855 healthy Norwegian women. PLoS One.

[19] Sun X, Cao ZB, Taniguchi H, Tanisawa K, Higuchi M (2017). Effect of an acute bout of endurance exercise on serum 25(OH)D concentrations in young adults. J Clin Endocrinol Metab.

[20] Seidell JC, Halberstadt J (2015). The global burden of obesity and the challenges of prevention. Ann Nutr Metab.

[21] Vainionpää A, Korpelainen R, Väänänen HK, Haapalahti J, Jämsä T, Leppäluoto J (2009). Effect of impact exercise on bone metabolism. Osteoporos Int.

[22] Maïmoun L, Simar D, Caillaud C, Peruchon E, Sultan C, Rossi M, Mariano-Goulart D (2008). Effect of antioxidants and exercise on bone metabolism. J Sports Sci.

[23] Kim H-J, Ji M, Song J, Moon H-W, Hur M, Yun Y-M (2017). Clinical utility of measurement of Vitamin D-binding protein and calculation of bioavailable Vitamin D in assessment of Vitamin D status. Ann Lab Med.

[24] Abboud M, Rybchyn MS, Ning YJ, Brennan-Speranza TC, Girgis CM, Gunton JE, Fraser DR, Mason RS. 1,25-dihydroxycholecalciferol (calcitriol) modifies uptake and release of 25-hydroxycholecalciferol in skeletal muscle cells in culture. J Steroid Biochem Mol Biol. 2017. 10.1016/j.jsbmb.2017.10.018 29107178

[25] Makanae Y, Ogasawara R, Sato K, Takamura Y, Matsutani K, Kido K, Shiozawa N, Nakazato K, Fujita S (2015). Acute bout of resistance exercise increases vitamin D receptor protein expression in rat skeletal muscle. Exp Physiol.

[26] Pojednic RM, Ceglia L, Olsson K, Gustafsson T, Lichtenstein AH, Dawson-Hughes B, Fielding RA (2015). Effects of 1,25-dihydroxyvitamin D3 and vitamin D3 on the expression of the vitamin d receptor in human skeletal muscle cells. Calcif Tissue Int.

[27] Rock CL, Emond JA, Flatt SW, Heath DD, Karanja N, Pakiz B, Sherwood NE, Thomson CA (2012). Weight loss is associated with increased serum 25-Hydroxyvitamin D in overweight or obese women. Obesity.

[28] Yeh JK, Aloia JF, Yasumura S (1989). Effect of physical-activity on calcium and phosphorus-metabolism in the rat. Am J Physiol.

[29] Baird J, Jacob C, Barker M, Fall CHD, Hanson M, Harvey NC, Inskip HM, Kumaran K, Cooper C (2017). Developmental origins of health and disease: a Lifecourse approach to the prevention of non-communicable diseases. Healthcare.

[30] Gilsanz V, Kremer A, Mo AO, Wren TA, Kremer R (2010). Vitamin D status and its relation to muscle mass and muscle fat in young women. J Clin Endocrinol Metab.

[31] Vainionpaa A, Korpelainen R, Leppaluoto J, Jamsa T (2005). Effects of high-impact exercise on bone mineral density: a randomized controlled trial in premenopausal women. Osteoporos Int.

[32] Maïmoun L, Mariano-Goulart D, Couret I, Manetta J, Peruchon E, Micallef JP, Verdier R, Rossi M, Leroux JL (2004). Effects of physical activities that induce moderate external loading on bone metabolism in male athletes. J Sports Sci.

[33] Zhu K, Whitehouse AJ, Hart PH, Kusel M, Mountain J, Lye S, Pennell C, Walsh JP (2014). Maternal vitamin D status during pregnancy and bone mass in offspring at 20 years of age: a prospective cohort study. J Bone Miner Res.

[34] Wong MWN, To WWK (2012). Bone mineral density changes during pregnancy in actively exercising women as measured by quantitative ultrasound. Arch Gynecol Obstet.

[35] Townsend N, Wilson L, Bhatnagar P, Wickramasinghe K, Rayner M, Nichols M (2016). Cardiovascular disease in Europe: epidemiological update 2016. Eur Heart J.

[36] Chin K, Zhao D, Tibuakuu M, Martin SS, Ndumele CE, Florido R, Windham BG, Guallar E, Lutsey PL, Michos ED (2017). Physical activity, Vitamin D, and incident atherosclerotic cardiovascular disease in whites and blacks: the ARIC study. J Clin Endocrinol Metab.

[37] Melzer K, Schutz Y, Boulvain M, Kayser B (2010). Physical activity and pregnancy: cardiovascular adaptations, recommendations and pregnancy outcomes. Sports Med.

[38] Zavorsky GS, Longo LD (2011). Exercise guidelines in pregnancy new perspectives. Sports Med.

[39] Gail MH, Benichou J, Armitage P, Colton T. Encyclopedia of epidemiologic methods: Wiley; 2000.

[40] Curran-Everett D, Milgrom H (2013). Post-hoc data analysis: benefits and limitations. Curr Opin Allergy Clin Immunol.

[41] Sainani KL (2009). The problem of multiple testing. PMR.

[42] Spiro A, Buttriss JL, Vitamin D (2014). An overview of vitamin D status and intake in Europe. Nutr Bull.

[43] Schwartz JB, Lai J, Lizaola B, Kane L, Markova S, Weyland P, Terrault NA, Stotland N, Bikle D (2014). A comparison of measured and calculated free 25(OH) vitamin D levels in clinical populations. J Clin Endocrinol Metab.

[44] Schulz KF, Grimes DA (2002). Sample size slippages in randomised trials: exclusions and the lost and wayward. Lancet.

[45] Fewtrell MS, Kennedy K, Singhal A, Martin RM, Ness A, Hadders-Algra M, Koletzko B, Lucas A (2008). How much loss to follow-up is acceptable in long-term randomised trials and prospective studies?. Arch Dis Child.

[46] Borodulin KM, Evenson KR, Wen F, Herring AH, Benson AM (2008). Physical activity patterns during pregnancy. Med Sci Sports Exerc.

[47] Hesketh KR, Evenson KR (2016). Prevalence of U.S. pregnant women meeting 2015 ACOG physical activity guidelines. Am J Prev Med.

[48] Gjestland K, Bo K, Owe KM, Eberhard-Gran M (2013). Do pregnant women follow exercise guidelines? Prevalence data among 3482 women, and prediction of low-back pain, pelvic girdle pain and depression. Br J Sports Med.

[49] da Silva SG, Hallal PC, Domingues MR, Bertoldi AD, Silveira MFD, Bassani D, da Silva ICM, da Silva BGC, Coll CVN, Evenson K (2017). A randomized controlled trial of exercise during pregnancy on maternal and neonatal outcomes: results from the PAMELA study. Int J Behav Nutr Phys Act.

