# Supplementary material for: The effect of an exercise program in pregnancy on vitamin D status among healthy, pregnant Norwegian women: a randomized controlled trial
Source: BMC Pregnancy Childbirth. 2019 Feb 20;19:76. doi: 10.1186/s12884-019-2220-z (PMC6381613; doi:10.1186/s12884-019-2220-z)
Supplement: Supplementary file 1 — Study protocol. (PDF 61 kb) [file 12884_2019_2220_MOESM1_ESM.pdf]

PROJECT PLAN

TRAINING DURING PREGNANCY

- **Effects of regular exercise during pregnancy in prevention of pregnancy-related diseases and complications during labour**

A randomised clinical trial

Signe Stafne Western
PhD-student
Dept of Community Medicine and General Practice, Norwegian University of Science and Technology, Trondheim, Norway

Siv Mørkved
PT, MSc, PhD
Senior Researcher Clinical Service, Hospital, Trondheim
Associate professor, Dept of Community Medicine and General Practice, Norwegian University of Science and Technology, Trondheim, Norway

Kjell Åsmund Salvesen
Professor MD, PhD
Department of Obstetrics and Gynecology, Norwegian University of Science and Technology, 7006 Trondheim, Norway.

BACKGROUND

Pregnancy is often regarded as a period of high risk when it comes to development of e.g. excessive weight gain, gestational diabetes and musculo-skeletal problems such as low back and pelvic girdle pain and urinary and fecal incontinence (Romem et al. 1991, Haugen 1998, Warren et al. 1997, Mørkved 2003).

While a pregnancy implies these and other risks, exercise is regarded as advantageous during the period, and is recommended in order to reduce negative symptoms, both physical and psychological (SEF 2000, ACOG 2003). Exercise is defined as regular, leisure-time physical activities, aimed improving physical condition, ability or health. Thus exercise must have a high enough level of intensity and regularity to improve the function of the heart and respiratory systems and muscles (Bouchard et al. 1993). Most published studies indicate that women with normal, uncomplicated pregnancies are fit for exercise with few restrictions, without the risk of hurting either themselves or their child (Sternfeld 1997, Riemann & Hansen 2000). In a review, Clapp (1996) concludes that regular exercise (30 min up to 5 days a week) is not negative for the fetus and has several positive effects on the mother.

Various epidemiological studies have examined the occurrence of different pregnancy-related diseases, and the relationship between such diseases and physical activity among pregnant women. While the pregnancy is a high-risk period for becoming overweight, the condition of being overweight during pregnancy is a risk factor for both the mother and the fetus (Baeten et al. 2001). Overweight in pregnancy is associated with fetal macrosomatia, and the combination is associated with prolonged labour, low apgar score, shoulder dystocia, nerve plexus injuries, increased proportion of instrumental deliveries and perineal ruptures Øian (2000). A greater increase in weight than 15 kilos implies a higher risk of developing disease in pregnancy and complications during labour (Haram et al. 1997), as well as problems with weight reduction after the birth (Rooney & Schauberger 2002). Some studies reveal that women who exercise before and during pregnancy weigh less, and are less likely to gain too much weight than those who do not (Clapp & Little 1995, Clapp 2000), while other studies conclude that exercise does not affect the weight of the mother. (Lokey et al. 1991, Sternfeld et al. 1995, Marquez-Sterling et al. 2000). These conflicting results are probably due to the difficulty of assessing the effect of exercise only, since weight gain is also dependent on food intake and other factors (Rössner 2000).

Gestational diabetes may be related to being overweight, and the prevalence varies between 5% and 12% (Baeten et al. 2001). There has been surprisingly little focus in scientific literature on the effects of physical activity during pregnancy in order to prevent gestational diabetes, despite the fact that the prevalence increases. The training of large muscle groups during pregnancy seems to have a positive effect on pregnancy-related diabetes, due to better insulin sensitivity and utilisation of glucose, and furthermore to a normalised level of blood sugar (Jovanovic-Peterson et al.1989, Garcia-Patterson et al. 2001). Dye et al. (1997) found that the effects of exercise might be positive when it comes to primary prevention of pregnancy-related diabetes. Exercise reduced the women's risk for developing pregnancy-related diabetes by 47 % among women with BMI>33.

Almost the half of all Norwegian women (42.4%) state that they have been troubled by low back / pelvic girdle pain during pregnancy (Endresen 1995). For many women, the pain

causes difficulties in their everyday work and life; for some of them, the experience from their pregnancy and postpartum period is dominated by pain, disability and reduced quality of life. The extent of these problems makes it a considerable problem not only for the women but also for society. Low back and pelvic girdle pain is the most common reason for sick leave for pregnant Swedish women (Østgaard et al. 1994a), and it has been reported that pregnancy-related pelvic girdle pain is the reason for approximately 300 000 days of sick leave per year in Denmark (Larsen et al. 1999).

Many women have continued pain in the lower back and pelvic girdle during the postpartum period (Mørkved et al. 1998, Larsen et al.1999), and about 1/5 of the women get chronic pain (Larsen et al. 1999, Østgaard et al. l997). There are several indications that the normal physiological changes during pregnancy lead to mechanical and structural changes which might cause low back and pelvic girdle pain during pregnancy and after delivery (Vleeming et al 1997). Most hypotheses about the cause of low back and pelvic girdle pain focus on changes in posture and reduced stability resulting from weight gain and hormonal influences. Several studies show changes in motor control strategies in women with pelvic girdle pain (Avery et al. 2000, O'Sullivan et al. 2002, Hungerford et al. 2003). Results from other studies indicate that exercise does have an effect on low back pain during pregnancy (Norén et al. 1997, Kihlstrand et al. 1999) and on pelvic girdle pain after delivery (Stuge et al. 2004). However, there is still uncertainty on the question of whether or not exercise during pregnancy can prevent pelvic girdle pain.

Pregnancy and childbirth are regarded as important risk periods in the development of incontinence. Prevalence of urinary incontinence varies between studies, with average prevalence estimates of 30-40% during pregnancy and the first months after childbirth (Mørkved & Bø 1999, Hunskaar et al. 2002, Mørkved 2003). Mørkved et al. (2003) found in a randomised clinical study that specific exercise of the pelvic floor muscles during pregnancy had effect on the prevention and treatment of urinary incontinence. A similar effect of pelvic floor muscle exercise has been reported in other studies, both during pregnancy (Reilly et al. 2002) and after childbirth (Mørkved & Bø 1997, Mørkved & Bø 2000). Prevalence of fecal incontinence after delivery is 4-5% (Mørkved & Bø 1999), and no RCT evaluating the effect of pelvic floor muscle exercise to prevent and treat fecal incontinence in a general pregnant and postpartum population has been published.

There is a myth that women who exercise regularly have stronger pelvic floor muscles that may obstruct labour (Riemann et al 2000), but there is no documentation for this claim (Lokey et al. 1991, Sternfeld et al. 1995, Horns et al. 1996). However, results from observational studies indicate that pregnant women who exercise regularly all through their pregnancy have more normal vaginal deliveries (Clapp 1990, Bungum et al. 2000, Clapp 2000), shorter active expulsion time and fewer complications during labour than those who do not exercise (Clapp 1990). In a randomised clinical trial, Salvesen and Mørkved (2004) found that fewer women who followed a scheme for specific strength exercise for the pelvic floor muscles during pregnancy had a prolonged second stage of labour, compared to a control group. Moreover, there was a lower prevalence of episiotomy and fewer cases of breech births in the exercise group (Salvesen & Mørkved 2004). This study is currently the only published randomised clinical trial which has tested such consequences of exercise before labour.

Today’s knowledge about the importance of exercise during pregnancy is mainly based on observational data from epidemiological studies, and the scientific strength of the clinical

recommendations given is open to question. There is a great lack of results from randomised clinical trials with high methodological quality, assessing the effects of exercise during pregnancy. As a result of this, many important questions are still not answered. One of these is the effect exercise during pregnancy has in the prevention and treatment of disease and complications which may arise during pregnancy. Another question is the consequences that exercise during pregnancy has for labour and delivery.

This study is thus designed to find answers to the following:

- **Does regular exercise during pregnancy aid in preventing gestational diabetes?**
- **Does regular exercise during pregnancy prevent low back and/or pelvic girdle pain?**
- **Does regular exercise during pregnancy prevent urine and/or fecal incontinence?**
- **Does regular exercise during pregnancy have an effect on labour and delivery?**
- **Does regular exercise during pregnancy prevent maternal excessive weight gain and fetal macrosomatia?**

MATERIAL AND METHODS

Subjects

Pregnant women who attend the routine ultrasound control at the three hospitals at 18 weeks of pregnancy are invited to participate in the study. Women are eligible for the trial if they are 18 years or more, with a singleton live foetus at the routine ultrasound scan. Exclusion criteria are pregnancy complications, high risk for preterm labour, pain during pelvic floor muscle contractions, ongoing urinary tract infection, or diseases that could interfere with participation (following recommendations from SEF 2000, ACOG 2003). In addition, women who live too far from the hospitals to be able to attend weekly exercise groups will be excluded. A total of 800 women will be included. A 12 months inclusion period is planned, in accordance with experiences from previous studies where 20% of eligible pregnant women agreed to participate in the trial (Mørkved & Bø 1997, Mørkved et al 2003).
The procedures to be followed will be in accordance with the ethical standards of the responsible regional committee on human experimentation and with the Helsinki declaration.

Design

We are planning a randomised clinical trial (RCT) with two arms; one group will attend a standardised regular exercise course for 12 weeks during pregnancy (Mørkved et al. 2003) (exercise group); another group will follow the standard procedure (control group). The trial is a multi-center study with blocked design, involving three hospitals in Trøndelag and Møre and Romsdal where a total of approximately 5000 women give birth annually (St.Olavs Hospital, Levanger Hospital and Ålesund Hospital). Participants at each hospital will be randomised to an exercise group or a control group. All participants will receive information concerning nutrition. Measurements will be taken of all participants before and after the intervention period during pregnancy, and 8 weeks and 1 year after delivery.

Randomisation procedure

A computerised randomisation procedure will be used.

Power calculation
The power calculations will be made taking into account the diseases we want to treat/prevent with the lowest prevalence (faecal incontinence and gestational diabetes (Mørkved & Bø 1999, Baeten et al 2001). We aim at reducing the prevalence from 5% to 1% in the exercise group. Based on these assumptions a two way t-test with a 5% level of significance and test strength of 0.90 give a study population of approximately 400 patients in each group (Altman 1991).

Evaluation methods
According to the aims of study, we have several primary outcome measures.

Several variables are routinely registered during pregnancy and labour:
During pregnancy
- Weight, height (BMI)

Labour and delivery
- Mode of delivery
- Epidural analgesia or oxytoxin augmentation during labour
- Episiotomy
- Perineal tears
- Neonatal outcomes
- Duration of 1. and 2. stages of labour

Other variables are registered by validated clinical measurement tools and/or questionnaires for registration of the women's own experiences:
- ***Gestational diabetes:***
  Oral glucose tolerance test at around 26 weeks
- ***Low back and pelvic girdle pain***
  Pain intensity 100mm Visual Analogue Scale
  Disability Rating Index (Salèn et al. 1994)
  Registration of sick leaves
  Clinical tests
    Active straight leg raise (ASLR) (Mens et al. 2000)
    Posterior pain provocation test (P4) (Kristiansson et al. 1996, Østgaard et al. 1994b)
    Balance (variations)
- ***Incontinence (urinary/faecal)***
  Self-reports of urinary/faecal incontinence. (Women reporting urinary/faecal incontinence once per week or more during the previous month are categorised as incontinent)
  Change in continence status (better, unchanged, worse)
  Strength and function of the pelvic floor muscles
  Vaginal palpation and observation during contraction used to assess the women's ability to perform pelvic floor muscle contraction. Pelvic floor muscle strength (vaginal squeeze pressure, cm H_2O) measured by a vaginal balloon catheter (balloon size 6.7 x 1.7 cm) connected to a pressure transducer (Camtech Ltd. 1300 Sandvika,

Norway). The method was found to be reliable and valid in a previous study (Bø et al 1990a).

- ***Registration of nutrition***
  Questionnaire (Nord-kost) (Nes et al. 1992).

Statistical analysis
The principal analysis will be done on an intention-to-treat basis. The missing last values are carried forward by their baseline values. Groups are compared with exact computation of Pearson χ^2 - test if data are categorical. Relative risks and their 95% confidence intervals are calculated for comparisons of proportions [observed ratio of proportions (StatXact -5)]. Normality will be evaluated by using the Shapiro-Wilk W test for normality, and the Mann-Whitney U test will be used to compare distributions between groups when variables are not normally distributed (SPSS-10). The influence of covariates on the primary outcome variables will be explored using logistic regression for odds ratio (SPSS-10). Additional subgroup analyses will be carried out. Results will be given as mean values with 95% confidence intervals (CI). P-values < 0.05 will be considered significant.

Interventions
Before randomisation, a physiotherapist will provide all women with information related to food intake and with individual instruction in pelvic floor anatomy and how to contract the pelvic floor muscles correctly (Bø et al 1990a).

The exercises group will follow a specially designed exercise course including specific exercises for stabilisation of the lower back and pelvis, the pelvic floor muscles, and general exercises including balance exercises. They will exercise with a physiotherapist for 60 minutes once a week for a period of 12 weeks (between 20 and 36 pregnancy weeks). In addition, the women will be encouraged to follow a home exercise program including 45 minutes of exercise twice a week (30 minutes endurance exercise and 15 minutes strength/balance exercise) and daily pelvic floor muscle contractions. Motivation will be strongly emphasised by the physiotherapists. Adherence to the training protocol will be based on registrations in the women's personal training diary and the reports from the physiotherapists who lead the group exercises. The training protocol follows recommendations from SEF (2000) and ACOG (2003).

Women in the control group will receive the customary information provided by their midwife or general practitioner. They will not be discouraged from exercising on their own.

Research group
Project manager and adviser for PhD students:
- Associate professor/Senior researcher, PhD Siv Mørkved, Dep. of Community Medicine and General Practice, NTNU, Trondheim / Clinical Services, St. Olavs Hospital, Trondheim

Medical responsible and adviser for PhD students:
- Professor Kjell Åsmund Salvesen, Dep Obstetrics and Gynecology, St. Olavs Hospital, Trondheim

Statistical analysis:

- Researcher, PhD Pål Romundstad, Dep. of Community Medicine and General Practice, NTNU, Trondheim

Data acquisition, analysis and publication (in cooperation with advisors):

- Two PhD students with doctoral fellowships

Responsible for information related to nutrition:

- Clinical nutrition physiologist, PhD student Ingrid Løvold Mostad, Clinical Services, St. Olavs Hospital, Trondheim

Exercise groups: 2 physiotherapists at St. Olavs Hospital, 1 physiotherapist at Levanger Hospital, 1 physiotherapists at Ålesund Hospital

Feasability

In both national and international literature the importance of physical activity are highlighted. WHO has recently presented a global strategy for nutrition, physical activity and health (Sosial- og helsedepartementet, 2004). In Norway physical activity and exercise have been strongly addressed, and is an issue of high priority also in pregnant and postpartum women (St.meld.nr.16, Handlingsplan for fysisk aktivitet). Nevertheless, few trials evaluating the effects of regular exercise have been published. The present trial aims at answering some questions related to the effects of regular exercise in a very important group, that is pregnant women.

Intervention trials including exercise programs are complicated, and needs close follow up of the participants. However, this research group has previously carried through several similar RCT's in women during pregnancy and after delivery (see publication lists).

REFERENCES

ACOG (American College of Obstetricians and Gynecologists).Exercise during pregnancy and the postpartum period. Clinical Obstetrics and Gynecology 2003; 46 (2): 496-499

Altman DG. Practical statistics for medical research. 1st edition 1991. Chapman and Hall, London, United Kingdom.

Baeten JM, Bukusi EA, Lambe M**.** Pregnancy complications and outcomes among overweight and obese nulliparous women. Am J Public Health 2001; 91:436

Bouchard C, Shephard RJ, Stephens T. Physical Activity, Fitness, and Health: Consensus Statement. 1993 Champaign: Human Kinetics Publishers

Bungum TJ, Peaslee DL, Jackson AW, Perez MA. Exercise during pregnancy and type of delivery in nulliparae. Journal of Obstetric, Gynecologic, and Neonatal Nursing 2000; 29(3): 258-264

Bø K, Hagen RH, Kvarstein B,Larsen S. Pelvic floor muscle exercise for the treatment of female stress urinary incontinence II. Validity of vaginal pressure measurements of pelvic floor muscle strength. The necessity of supplementary methods for control of correct contraction. Neurourol Urodyn 1990a;9:479-87.

Bø K, Hagen RH, Kvarstein B, Jørgensen J, Larsen S. Pelvic floor muscle exercise for the treatment of female stress urinary incontinence. III. Effects of two different degrees of pelvic floor muscle exercises. Neurourol Urodyn 1990b;9:489-502.

Bø K. Reproducibility of instruments designed to measure objective evaluation of female stress urinary incontinence. Scand J Urol Nephrol 1994;28:97-100.

Clapp JF III. The course of labor after endurance exercise during pregnancy. American Journal of Obstetrics and Gynecology 1990; 163: 1799-1805

Clapp JF III, Little KD. Effect of recreational exercise on pregnancy weight gain and subcutaneous fat deposition. Medicine and Science in Sports and Exercise 1995; 27(2): 170-177

Clapp JF III. Morphometric and neurodevelopmental outcome at age five years of the offspring of women who continued to exercise regulary throughout pregnancy. The Journal of Pediatrics 1996; 129(6): 856-863

Clapp JF III. Exercise during pregnancy. A clinical update. Clinics in Sports Medicine 2000; 19(2): 273-286

Dye TD, Knox KL, Artal R, Aubry RH, Wojtowycz (1997). Physical activity, obesity, and diabetes in pregnancy. American Journal of Epidemiology 1997; 146: 961-965

Endresen, E. H. Pelvic pain and low back pain in pregnant women-an epidemiological study. Scand.J Rheumatol. 1995; 24(3): 135-141

Garcia-Patterson A, Martin E, Ubeda J, Maria MA, De Leiva A & Corcoy R. (2001). Evaluation of light exercise in the treatment of gestational diabetes. Diabetes Care 2001; 24(11): 2006-2007

Haram K, Bergsjø P, Tangvik RJ. Vekt og vektøkning hos gravide. Tidsskrift for Den Norske Lægeforening 1997; 117(22): 3230-3233

Haugen IE. Svangerskap, fødsel og barseltid, 6. utg. 1998 Oslo: Ad Notam Gyldendal.

Handlingsplan for fysisk aktivitet 2005-2009; Helse- og omsorgsdepartementet (Sammen for fysisk aktivitet).

Horns, PN, Ratcliffe LP, Leggett JC, Swanson MS. Pregnancy outcomes among active and sedentary primiparous women. Journal of Obstetric, Gynecologic, & Neonatal Nursing 1996; 25(1): 49-54

Hungerford B, Gilleard W, Hodges P. Evidence of altered lumbopelvic muscle recruitment in the presence of sacroiliac joint pain. Spine 2003; 28(14): 1593-1600

Hunskaar S, Burgio K, Diokno AC, Herzog AR, Hjälmås K, Lapitan MC. Epidemiology and natural history of urinary incontinence (UI). Page 167. In: Abrams P, Cardozo L, Khoury S, Wein A. Incontinence. 2nd International Consultation on Incontinence July1-3, 2001. 2nd Edition 2002.Plymbridge Distributors Ltd, Plymbridge, United Kingdom.

Jovanovic-Peterson, L., Durak, E. P. & Peterson, C. M. (1989). Randomized trial of diet versus diet plus cardiovascular conditioning on glucose levels in gestational diabetes. American Journal of Obstetrics and Gynecology, 161: 415-419

Kihlstrand M. Stenman B. Nilsson S. Axelsson O. Water-gymnastics reduced the intensity of back/low back pain in pregnant women. Acta Obstetricia et Gynecologica Scandinavica 1999; 78(3): 180-185

Kristiansson P, Svardsudd K, & von Schoultz B. 1996 Back pain during pregnancy: a prospective study. Spine 21(6): 702-709

Larsen EC, Wilken-Jensen C, Hansen A, Jensen DV, Johansen S, Minck H, Wormslev M, Davidsen M, Hansen T M. Symptom-giving pelvic girdle relaxation in pregnancy. I: Prevalence and risk factors. Acta Obstet.Gynecol.Scand. 1999;78(2): 105-110

Lokey E A, Tran ZV, Wells CL, Myers BC, Tran AC. Effects of physical exercise on pregnancy outcomes: a meta-analytic review. Medicine and Science in Sports and Exercise 1991; 23(11): 1234-1239

Marquez-Sterling S, Perry AC, Kaplan TA, Halberstein RA, Signorile JF (2000). Physical and psychological changes with vigorous exercise in sedentary primigravidae. Medicine & Science in Sports & Exercis 2000; 32(1): 58-62

Mørkved S, Bø K. The effect of postpartum pelvic floor muscle exercise in the prevention and treatment of urinary incontinence. Int Urogynecol J 1997;8:217-222.

Morkved, S. Prevalence of pelvic girdle pain during pregnancy and postpartum. 3rd interdisciplinary world congress on low back and pelvic pain. The most effective role for exercise therapy, manual techniques, surgery and injection techniques. 1998 edn, European Conference Organizers, Rotterdam 427-428

Mørkved S, Bø K . Prevalence of urinary incontinence during pregnancy and postpartum. Int Urogynecol J 1999;10:394-398.

Mørkved S, Bø K. Effect of postpartum pelvic floor muscle training in prevention and treatment of urinary incontinence: a one-year follow up. Br J Obstet Gynaecol 2000; 107:1022-1028.

Mørkved S, Bø K, Schei B, Salvesen KÅ. Pelvic floor muscle training during pregnancy to prevent urinary incontinence - a single blind randomized controlled trial. Obstetrics & Gynecology 2003;101:313-319.

Mørkved S. Urinary incontinence during pregnancy and after delivery. - Effect of pelvic floor muscle training in prevention and treatment. Dr.philos. thesis 2003. Department of Community Medicine & General practice, National Center for Fetal Medicine, Department of Laboratory Medicine and Children`s and Women`s Health, Faculty of Medicine, Norwegian University of Science and Technology.

Nes M, Frost Andersen L, Solvoll K, Sandstad B, Hustvedt BE, Lovo A, Drevon CA. Accuracy of a quantitative food frequency questionnaire applied in elderly Norwegian women. Eur J Clin Nutr. 1992 Nov;46(11):809-21

Norén, L., Östgaard, S., Nielsen, T. F. & Östgaard H.C. Reduction of sick leave for lumbar back and posterior pelvic pain in pregnancy. Spine 1997; 22 (18): 2157-2160

O`Sullivan, P., Twomey, L., Allison, G., Sinclair, J., Miller, K., & Knox, J. 1997 Altered patterns of abdominal muscle activation in patients with chronic low back pain, Australian Journal of Physiotherapy 43: 91-98

Reilly ETC, Freeman RM, Waterfield MR, Waterfield AE, Steggles, Pedlar F. Prevention of postpartum stress incontinence in primigravidae with increased bladder neck mobility: a randomised controlled trial of antenatal pelvic floor exercises. BJOG 2002;109:68-76.

Riemann M K, Hansen I-L K. (2000). Effects on the foetus of exercise in pregnancy. Scandinavian Journal of Medicine & Science in Sports, 10: 12-19

Romem Y, Masaki DI, Artal R. Physiological and endocrine adjustments to pregnancy. I: Artal, R., Wiswell, R. A. & Drinkwater, B. L. (Red.), Exercise in pregnancy, 2. utg. 1991. Baltimore: Williams & Wilkins

Rooney BL, Schauberger CW. (2002). Excess pregnancy weight gain and long-term obesity: one decade later. Obstetrics and Gynecology 2002; 100 (2): 245-252

Rössner S. Physical activity and prevention and treatment of weight gain associated with pregnancy: current evidence and research issues. I: Bouchard Claude (Red.), Physical activity and obesity. 2000 Champaign: Human Kinetics

Salvesen KÅ, Mørkved S. Randomised controlled trial of pelvic floor muscle training during pregnancy. BMJ 2004;329:378-80.

Salèn BA, Spangfort EV, Nygren ÅL, Nordemar R. 1994 The Disability Rating Index: An Instrument for the Assessment of Disability in Clinical Settings. J Clin Epidemiol. . 47(12): 1423-1434

SEF (Statens råd for ernæring og fysisk aktivitet) (2000). Fysisk aktivitet og helse. Anbefalinger. Rapport nr. 2/2000. Lokalisert 17.12.2004 på Verdensveven: http://www.sef.no/assets/11000147/rapportferdig.pdf

Sosial- og helsedepartementet, Strategi for kosthold, fysisk aktivitet og helse, 2004

Sternfeld B, Quesenberry CP Jr, Eskenazi B, Newman LA. Exercise during pregnancy and pregnancy outcome. Medicine & Science in Sports and Exercise 1995; 27(5): 634-640

Sternfeld, B. Physical Activity and Pregnancy Outcome. Sports Medicin 1997;, 23(1): 33-47

St.meld. nr.16 (2002-2003) Resept for et sunnere Norge – Folkehelsepolitikken (Folkehelsemeldingen)

Stuge B, Lærum E, Kirkesola G, Vollestad N (2004). The efficacy of a treatment program focusing on specific stabilising exercises for pelvic girdle pain after pregnancy: a randomized controlled trial. Spine, 29 (4): 351-359

Vleeming A, Snijders CJ, Stoeckart R, Mens JMA. The role of the sacroiliac joints in coupling between spine, pelvis, legs and arms, in Movement, stability & low back pain. The essential role of the pelvis, First edn, 1997. A. Vleeming et al., eds., Churchill Livingstone, The Netherlands, 53-71

Warren MP, Shangold, MM. Sports Gynecology: Problems and Care for the Athletic Female. 1997 Cambridge: Blackwell Science

Øian P. Tidskr. Nor Lægeforen. 2000;120:1847

Østgaard HC, Zetherstrøm G, Roos-Hansson E. The posterior pelvic pain provocation test in pregnant women. Eur Spine J 1994;3:258-260.

Ostgaard HC, Zetherstrom G, Roos-Hansson E, Svanberg B. Reduction of back and posterior pelvic pain in pregnancy. Spine 1994a;19(8): 894-900

Ostgaard HC, Zetherstrom G, Roos-Hansson E. The posterior pelvic pain provocation test in pregnant women. Eur.Spine J. 1994b; 3(5): 258-260

Ostgaard HC, Zetherstrom, G, Roos-Hansson E. Back pain in relation to pregnancy: a 6-year follow-up. Spine1997; 22(24): 2945
