# Supplementary material for: The effect of an exercise program in pregnancy on vitamin D status among healthy, pregnant Norwegian women: a randomized controlled trial
Source: BMC Pregnancy Childbirth. 2019 Feb 20;19:76. doi: 10.1186/s12884-019-2220-z (PMC6381613; doi:10.1186/s12884-019-2220-z)
Supplement: Supplementary file 2 — The reference range, limit of detection and total analytical coefficient of variation (CV) of biochemical methods used. (PDF 61 kb) [file 12884_2019_2220_MOESM2_ESM.pdf]

Additional file 2. The reference range, limit of detection and total analytical coefficient of variation (CV) of biochemical methods used

**Serum measure**	**Method for measurement (manufacturer)**	**Total analytical coefficient of variation** (CV)	**The limit of detection**	**Reference range**
Total 25(OH)D (nmol/L)[*]	ECLIA (Roche Diagnostics Ltd)	8.6% at 70 nmol/L	8 nmol/L	45-161 nmol/L
PTH (pmol/L)[*]	ECLIA (Roche Diagnostics Ltd)	3.5% at 2.7 pmol/L	0.1 pmol/L	1.6-6.9 pmol/L
Total calcium (mmol/L)[*]	Colorimetric method (Roche Diagnostics Ltd)	1.5% at 2.6 mmol/L	0.20 mmol/L	2.15-2.51 mmol/L
Magnesium (mmol/L)[*]	Photometric method (Roche Diagnostics Ltd)	1.6% at 0.43 mmol/L	0.03 mmol/L	0.71-0.94 mmol/L
Phosphate (mmol/L)[*]	Photometric method (Roche Diagnostics Ltd)	1.9% at 1.1 mmol/L	0.1 mmol/L	0.85-1.50 mmol/L
Albumin (g/L)[*]	Photometric method (Roche Diagnostics Ltd)	1.7% at 45 g/L	5 g/L	18-39 years old: 36-48 g/L<br>40-46 years old: 36-45 g/L
DBP (µmol/L)[**]	RIA with polyclonal antibody (Hormone Laboratory, Oslo University Hospital)	13% at 6.8 µmol/L	0.7 µmol/L	2. trimester: 4.6-11.28 µmol/L<br>3. trimester: 3.38-12.9 µmol/L

[*]25(OH)D, PTH, total calcium, magnesium, phosphate and albumin were analyzed at Department of Medical Biochemistry, St. Olavs hospital, Trondheim University Hospital.

[**]DBP was analyzed at Hormone Laboratory, Oslo University Hospital.

Abbreviations: CV, total analytical coefficient of variation; PTH, parathyroid hormone; ECLIA, electrochemiluminescence immunoassay; DBP, Vitamin D-binding protein; RIA, radioimmunoassay.
