# Supplementary material for: The effect of an exercise program in pregnancy on vitamin D status among healthy, pregnant Norwegian women: a randomized controlled trial
Source: BMC Pregnancy Childbirth. 2019 Feb 20;19:76. doi: 10.1186/s12884-019-2220-z (PMC6381613; doi:10.1186/s12884-019-2220-z)
Supplement: Supplementary file 3 — Calculation of free and bioavailable 25(OH)D. (PDF 469 kb) [file 12884_2019_2220_MOESM3_ESM.pdf]

Additional file 3. Calculation of free and bioavailable 25(OH)D

Free 25(OH)D is presented according to Bikle et al.:

$$\mathrm{D_{free}} = \frac{\mathrm{D_{total}}}{\big(1 + ([\text{binding constant albumin}] \times \text{albumin}) + ([\text{binding constant DBP}] \times \text{DBP})\big)}$$

D_{free} is the calculated free levels of 25(OH)D. D_{total} is the total serum levels of 25(OH)D. Albumin-bound 25(OH)D (D_{alb}) was calculated as follows:

$$\mathrm{D_{alb}} = (\text{free 25(OH)D} \times [\text{binding constant albumin}] \times \text{albumin})$$

The binding constant was 6 x10^5 M^{-1} between 25(OH)D and albumin. The binding constant was 7 x 10^8 M^{-1} between 25(OH)D and DBP.

Bioavailable 25(OH)D was calculated as the sum of albumin-bound and free 25(OH)D.
